# Supplementary material for: Complex pathways and memory in compressed corrugated sheets
Source: Proc Natl Acad Sci U S A. 2021 Dec 7;118(50):e2111436118. doi: 10.1073/pnas.2111436118 (PMC8685682; doi:10.1073/pnas.2111436118)
Supplement: Supplementary File [file pnas.2111436118.sapp.pdf]

PNAS
www.pnas.org

Supplementary Information for

Complex pathways and memory in compressed corrugated sheets

Hadrien Bense, Martin van Hecke

Hadrien Bense.
hbense.h@gmail.com

This PDF file includes:

Supplementary text
Figs. S1 to S3 (not allowed for Brief Reports)
Legends for Movies S1 to S3

Other supplementary materials for this manuscript include the following:

Movies S1 to S3

Supporting Information Text

Supplementary Videos 1, 2 and 3 Front view of sample A during a compression/decompression cycle presenting the main loop for three increasing values of the tilt angle α. These three movies demonstrate how the order of snapping and unsnapping events is modified by the tilt angle: supplementary movie 1 corresponds to topology A(i), supplementary movie 2 to A(vi) and supplementary movie 3 to A(vii). The compression speed is 5 mm/min, the movies were recorded at a frame rate of 24Hz, and are real time.

Reproducibility, plasticity and ageing We performed 150 compression cycles on sample A, where each driving sub-cycle (Fig. 1i, inset) is repeated 50 times, and observe a very good reproducibility of the pathways and response (Supplementary Fig. S1a). We also monitored the force relaxation under large compression, and find a force decay of approximately 7% over three hours (Supplementary Fig. S1b). We estimate the amount of relaxation during typical experiments (compression is close to the maximum for approximately 1 min) to be on the order of 4%.) While such relaxation has a minor impact on the exact values of the switching fields, it does not influence the topology of the t-graphs. Moreover, such relaxation is fully reversible after releasing the load, and long-term plastic ageing of the sample is essentially absent: the data presented in this paper were acquired over the course of many months, with no visible changes in the mechanical response. Similarly, we emphasize the high reproducibility of the mechanical response when a tilt angle is revisited, and have been able to easily reproduce the full response is such cases (Supplementary Fig. S1c-d).

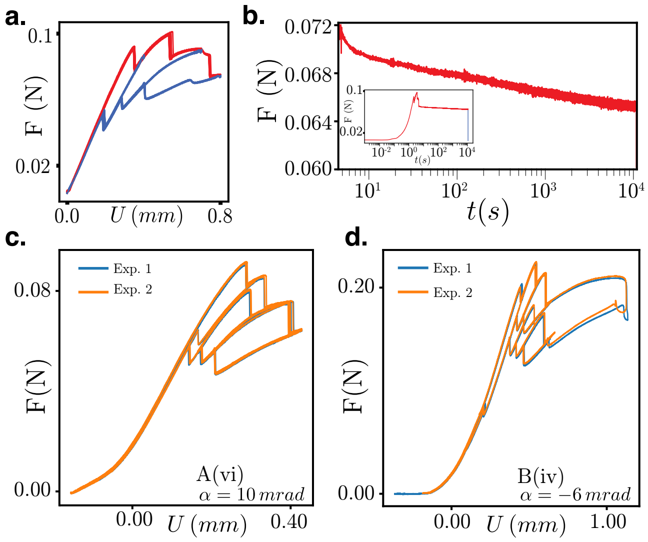

Fig. S1. **a**, 150 cycles of compression performed on sample A (protocol similar as inset of Fig. 1i, where each sub-cycle is repeated 50 times). Deformation pathways reproduce. **b**, Force relaxation of sample A. Compression is maintained at $U = 0.8$ mm for three hours, the force relaxes by approximately 7%, with no creeping effect. Inset: full signal **c,d** When a tilt angle α is revisited, the mechanical response reproduces (c: sample A; d: sample B).

Sample B. We perform a systematic tilting experiment with the four grooves sample B, whose other geometrical parameters are close to sample A (Supplementary Fig. S2). We obtained nine t-graphs, eight of which are Preisach, and one is scrambled (Supplementary Fig. S2a1, a2). Similar to sample A (Fig. 2), most changes in the topology of the t-graphs are associated with a single permutation of P^+ or P^- (Supplementary Fig. S2a1, a2). We attribute the $B(i)$ to $B(ii)$ and $B(vi)$ to $B(vii)$ cases, that involve two permutations, to the near degeneracy of two t-graph transitions. A close inspection of the switching fields reveals that a change in the t-graphs topology coincides with the crossing of the relevant switching fields (Supplementary Fig. S3b1,b2). Finally, despite the very weak interactions (as seen by the very small state dependence of the various switching fields, Supplementary Fig. S3b1,b2), we found a t-graph B(v) whose transitions $\{1111\} \to \{1011\}$ and $\{0111\} \to \{0101\}$ are scrambled. B(v) exists on a very narrow angle range (Supplementary Fig. S2b), where the relevant switching fields are quite close, but we nevertheless have been able to reproduce it several times.

Switching fields. Figure S3 presents the different switching fields as a function of the tilt angle α, for all samples presented in the paper.

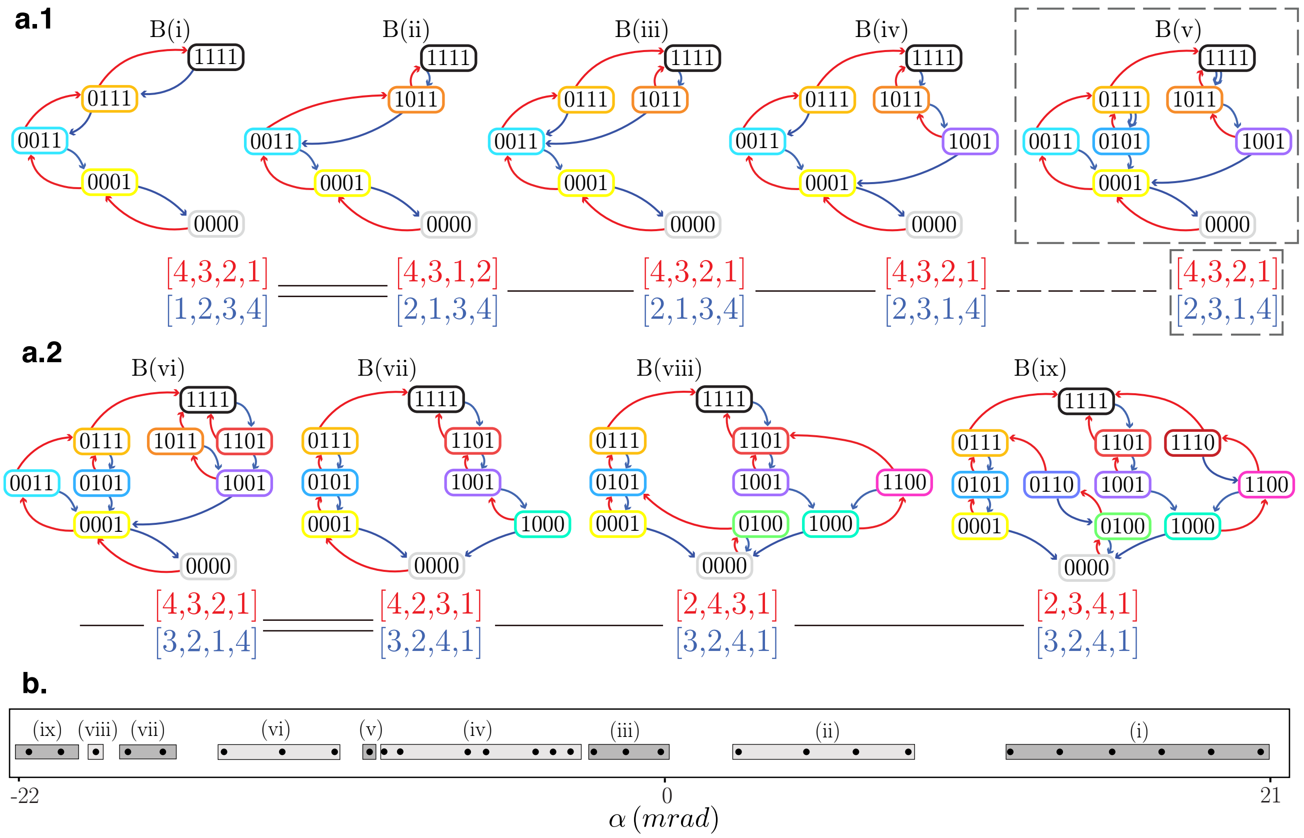

Fig. S2. Tunable Preisach transition graphs (sample B). a1,2, Tilting the bottom boundary of sample B ($\{H, t, R, p, A_0\} = \{30, 0.3, 1.0, 8, 3\}$ mm and $N = 4$ corrugations) elicits nine Preisach t-graphs and one scrambled t-graphs (dashed box), where the scrambled transitions are shown by double arrows. The red (blue) lists indicate the order of the up (down) transitions of the main loop P^+ (P^-).The angle α increases monotonically from B(ix) to B(i) **b**, t-graph as function of the tilt angle α.

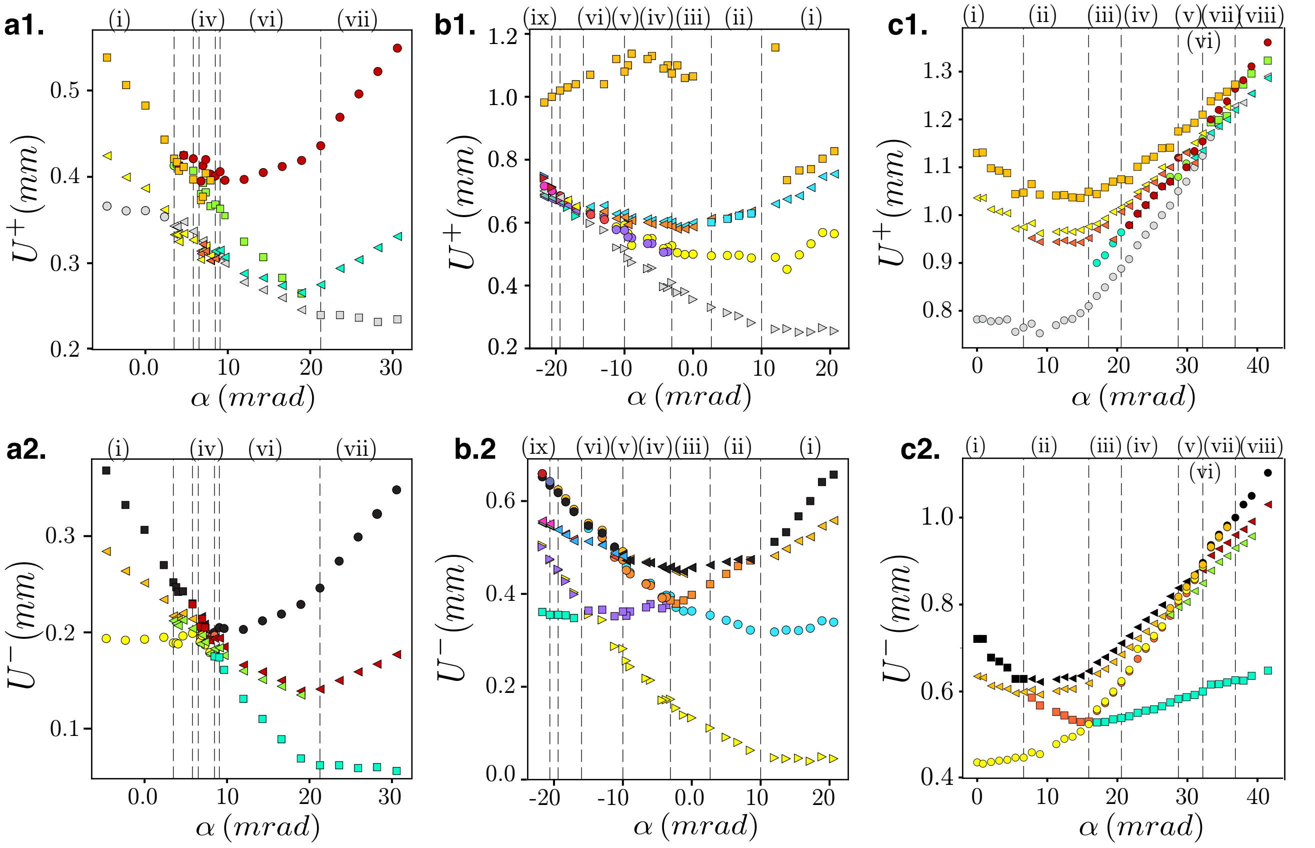

Fig. S3. Switching fields. U^+ and U^- as a function of the tilt angle α (**a**: sample A, **b**: sample B, **c**: sample C). Symbols refer to hysteron 1 (□), 2 (◁), 3 (○) or 4 (▷); colors refer to the state. For example, a yellow circle in panel (b1) shows $U_3^+(0001)$. Dotted lines mark the boundaries between network topologies, with some narrow ranges not labeled for legibility.

Movie S1. Topology $A(i)$

Movie S2. Topology $A(vi)$

Movie S3. Topology $A(vii)$
